# Ropinirole suppresses LPS-induced periodontal inflammation by inhibiting the NAT10 in an ac4C-dependent manner

**DOI:** 10.1186/s12903-024-04250-5

**Published:** 2024-04-30

**Authors:** Haiqing Liao, Huabing Ma, Hongying Meng, Na Kang, Lufei Wang

**Affiliations:** Guangxi Key Laboratory of Oral and Maxillofacial Rehabilitation and Reconstruction & Guangxi Health Commission Key Laboratory of Prevention and Treatment for Oral Infectious Diseases & College and Hospital of Stomatology, Guangxi Medical University, No.10, Shuangyong Road, Nanning, 530021 Guangxi China

**Keywords:** Periodontitis, Human gingival fibroblasts, NAT10, ac4C, KLF6

## Abstract

**Background:**

Periodontitis is a chronic osteolytic inflammatory disease, where anti-inflammatory intervention is critical for restricting periodontal damage and regenerating alveolar bone. Ropinirole, a dopamine D2 receptor agonist, has previously shown therapeutic potential for periodontitis but the underlying mechanism is still unclear.

**Methods:**

Human gingival fibroblasts (HGFs) treated with LPS were considered to mimic periodontitis in vitro. The dosage of Ropinirole was selected through the cell viability of HGFs evaluation. The protective effects of Ropinirole on HGFs were evaluated by detecting cell viability, cell apoptosis, and pro-inflammatory factor levels. The molecular docking between NAT10 and Ropinirole was performed. The interaction relationship between NAT10 and KLF6 was verified by ac4C Acetylated RNA Immunoprecipitation followed by qPCR (acRIP-qPCR) and dual-luciferase reporter assay.

**Results:**

Ropinirole alleviates LPS-induced damage of HGFs by promoting cell viability, inhibiting cell apoptosis and the levels of IL-1β, IL-18, and TNF-α. Overexpression of NAT10 weakens the effects of Ropinirole on protecting HGFs. Meanwhile, NAT10-mediated ac4C RNA acetylation promotes KLF6 mRNA stability. Upregulation of KLF6 reversed the effects of NAT10 inhibition on HGFs.

**Conclusions:**

Taken together, Ropinirole protected HGFs through inhibiting the NAT10 ac4C RNA acetylation to decrease the KLF6 mRNA stability from LPS injury. The discovery of this pharmacological and molecular mechanism of Ropinirole further strengthens its therapeutic potential for periodontitis.

## Introduction

Periodontitis is a chronic inflammation. Its clinical manifestations are gingival bleeding, periodontal pocket formation, and alveolar bone resorption. Previous studies have shown that the process of alveolar bone resorption in periodontitis is related to the activation of NF-κB signaling pathway on osteoclast cell membrane by binding of various inflammatory factors to their corresponding receptors [1]. The treatment methods for periodontitis in clinical practice include periodontal initial therapy, drug therapy and periodontal surgery, but these methods still have limitations, such as unable to effectively reduce the expression level of inflammatory factors in periodontal tissue and drug side effects [2, 3]. Therefore, it is of vital significance to design a treatment plan that treats both the symptoms and root causes of periodontitis, and it is also necessary to find drugs to reduce the inflammation of periodontitis.

Non-steroidal anti-inflammatory drugs have been reported to improve the clinical outcome of mechanical periodontal treatment due to the effects of alveolar bone resorption inhibition [4]. Four-octyl itaconate treatment ameliorates inflammation induced by LPS in the periodontal microenvironment [5]. Stevioside reduces inflammation in periodontitis by changing the oral bacterial composition [6]. At present, there are some studies using antimicrobials such as amoxicillin, metronidazole, and azithromycin to assist the treatment of periodontitis [7]. Therefore, it is of significance to search for potential drugs for periodontitis treatment. Ropinirole hydrochloride, a dopamine D2 receptor agonist, is commonly used for Parkinson’s disease [8, 9]. Previous studies have shown that Ropinirole inhibits the expression of CXCL1 in gingival epithelial cells through dopamine-like receptors, thereby inhibiting neutrophil inflammation and alveolar bone destruction in a murine periodontitis model [10]. Inhibiting inflammation can promote alveolar bone regeneration and repair to treat periodontitis [11]. Therefore, Ropinirole may be a promising drug for the treatment of periodontitis. However, the underlying mechanism needs to be further investigated.

N-acetyltransferase 10 (NAT10) is an acetyltransferase that has been shown to catalyze N4‐acetylcytidine (ac4C) mRNA modification and has been implicated in cellular pathophysiological processes, including inflammatory immune responses [12, 13]. Studies have shown that the expression of NAT10 in macrophages is reduced during LPS-induced inflammation, and NAT10 knockdown significantly reduces the production of inflammatory factors [13]. Therefore, we wanted to know whether Ropinirole in vitro treatment of periodontitis could be achieved by activating this mechanism. In this study, we evaluated the inflammatory factor levels of human gingival fibroblasts (HGFs) before and after Ropinirole treatment, and the NAT10-mediated ac4C modification regulated by Ropinirole was also studied.

## Materials and methods

### Collection and treatment of HGFs

HGFs purchased from ScienceCell were cultured in α-MEM (Hyclone) containing 10% FBS (Gibco) at 37 °C with 5% CO_2_ in a cell incubator. HGFs were then treated with 1 µg/mL lipopolysaccharide (LPS) from Porphyromonas gingivalis (ST1470; Beyotime) for 24 h after transfection [14]. In some experiments, HGFs were treated with Ropinirole (YZ-1,605,205; Solarbio) at the concentration of 25, 50, 100, 200, or 400 µg/ml prior to the addition of LPS. After 24 h of incubation, the cells and supernatants were harvested.

### Molecular docking

The molecular docking between NAT10 and Ropinirole was performed to verify the potential target. Protein crystal structure of NAT10 was downloaded from RCSB PDB database (https://www1.rcsb.org/) and the pdb file was obtained. The mol2 file of Ropinirole were downloaded from PubChem database (https://pubchem.ncbi.nlm.nih.gov/), and AutoDock Tools was used to process protein crystals and compound structures. Finally the files were converted into PDBQT format and molecular docking was conducted. The docking results are visualized by PyMOL software.

### In vitro transfection

The pcDNA3.1-NAT10 and pcDNA3.1-KLF6 expression plasmids were constructed by Shanghai Biological Engineering Company. HGFs were seeded in six-well plates (2 × 10^5^ cells / well) prior to transfection. The transfection was performed using Effectene Transfection Reagent (QIAGEN Companies) according to the manufacturer’s instructions when cell confluence reached to over 60% confluence. The experimental and control groups were termed pc-NAT10, pcKLF6 and pcDNA3.1, respectively.

As for NAT10 inhibition, the short hairpin RNA (shRNA) lentivirus of NAT10 (shNAT10) and negative control (shNC) lentivirus were constructed by GenePharma Co., LTD. HGFs were transfected with the specified vector using the Lipofectamine 3000 (Invitrogen) regeant for 48 h.

### Flow cytometry assay

Cell apoptosis was detected by an Annexin V-FITC Apoptosis Detection Kit (KeyGEN BioTECH). Briefly, HGFs were seeded in 6-well plates at 1 × 10^6^ cells/well. After collected and washed twice in cool PBS, HGFs were suspended in 100 µL 1× binding buffer. We added 5 µL Annexin V-FITC and 5 µL PI in the dark for 20 min incubation. Finally, 400 µL of 1× binding buffer was added to the cells. The percentage of positive cells was detected using CytoFLEX (Beckman Coulter) following the manufacturer’s instructions.

### Enzyme-linked immunosorbent assay (ELISA)

The supernatant was collected for the detection of inflammatory cytokines. Analysis was performed using a microplate reader, which measured absorbance at 450 nm, and commercially available ELISA kits for IL-1β (PI305), IL-18 (PI558), and TNF-α (PT518) according to the manufacturer’s instructions. Experiments were repeated two times with three biological replicates.

### Quantitative reverse transcription PCR (RT-qPCR) and ac4C acetylated RNA immunoprecipitation followed by qPCR (acRIP-qPCR)

RT-PCR was performed with the SuperScript ™II cDNA Synthesis Kit for qPCR (18,064,071; Thermo Fisher) and the SuperScript™ III Platinum™ SYBR™ Green PCR Kit (11,746,500; Invitrogen) on an Applied Biosystems 7500 sequence detection system with triplicate reactions. The primers used were listed in Table 1. As for acRIP-qPCR, RNA fragments were incubated with an anti‐ac4C antibody (ab252215; Abcam) or IgG (ab172730; Abcam), and ac4C‐modified RNA was then eluted with N‐acetylcytidine sodium salt for ac4C enrichment analysis by qPCR.

**Table 1:** The primer sequences for qRT-PCR

Primers	Sequences
GAPDH-Forward	CGGAGTCAACGGATTTGGTCGTAT
GAPDH-Reverse	AGCCTTCTCCATGGTGGTGAAGAC
NAT10-Forward	ATAGCAGCCACAAACATTCGC
NAT10-Reverse	ACACACATGCCGAAGGTATTG
KLF6-Forward	TCTCATCAGCCCGAGCTTTTG
KLF6-Reverse	GAGCTGTCAGAGGATTCGCT

### Actinomycin D treatment

Actinomycin D (2 mg/mL, Sigma) were used to test the mRNA stability of KLF6 measured by RT-qPCR.

### Dual luciferase reporter assay

The pGL3 luciferase promoter plasmid (Promega) was used to generate constructs containing KLF6 3′-UTR [defined as wildtype (wt)]. The KLF6 3′-UTR mutation point [defined as mutant-type (mut)] that containing ac4C sites were directly synthesized using the QuickChange Multiple Site-directed Mutagenesis Kit (Stratagene). Afterwards, all designed plasmids were transfected into HGFs using the Lipofectamine 3000 method (Invitrogen). Fourty-eight hours after transfection, luciferase activities of harvested HGFs were detected by the Dual Luciferase Reporter Assay Kit (Promega). The ratio of firefly to Renilla luciferase activity was subsequently determined.

### Statistical analysis

Statistical analysis was conducted using SPSS 24.0 (IBM Corp., USA). Data were demonstrated as mean ± SD. Student’s t-test (two groups) and one-way analysis of variance (ANOVO) followed by Tukey’s-test (> 2 groups) were applied for difference analysis. *P* value less than 0.05 was deemed statistically significant.

## Results

### Ropinirole alleviates LPS-induced damage of HGFs

**Fig. 1:**
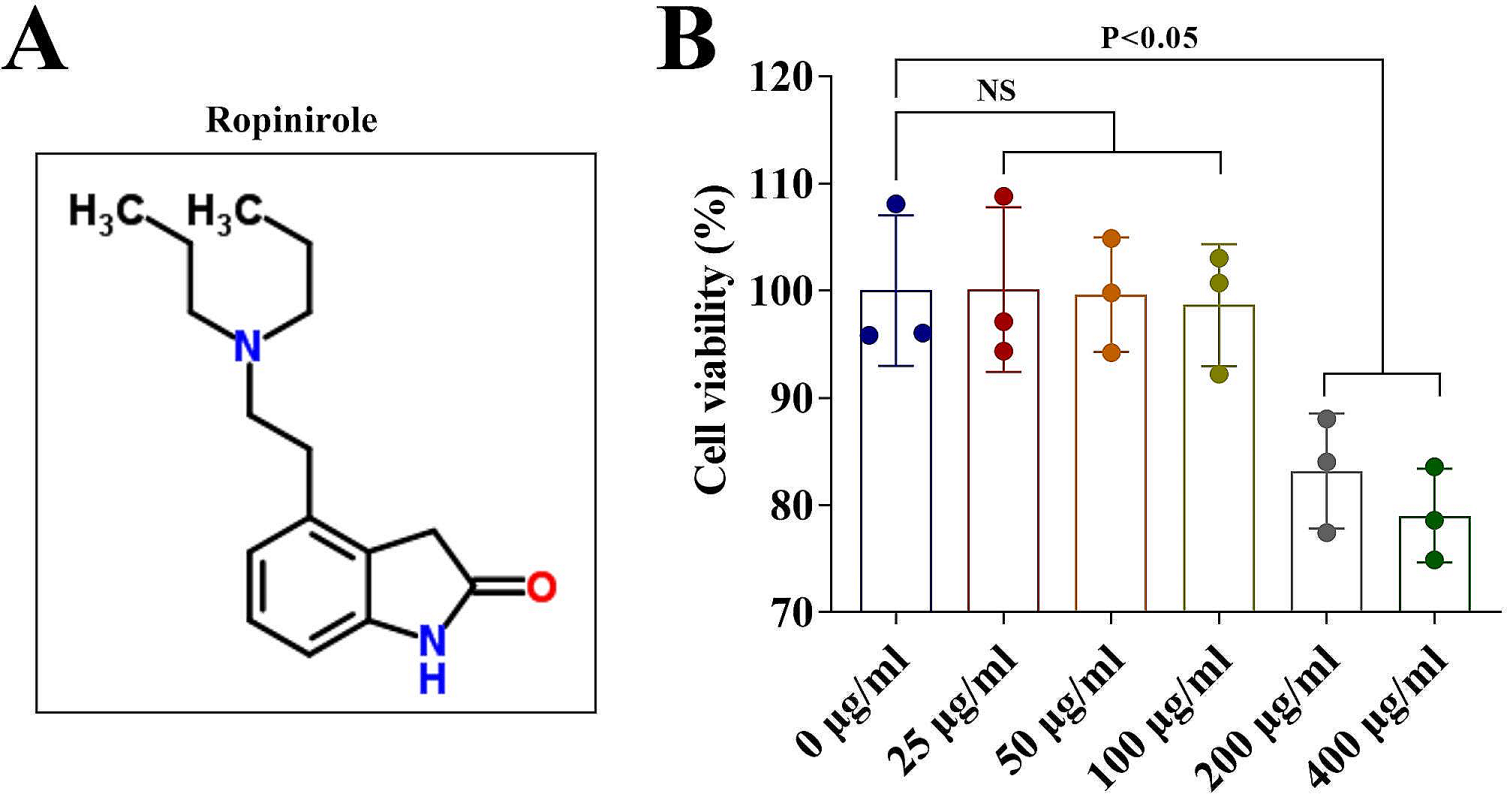
Ropinirole at concentrations of 25, 50 and 100 µg/ml had no statistically significant effect on the cell viability of HGFs. (**A**) The chemical structural formula of Ropinirole. (**B**) The cell viability of HGFs treated with different concentration of Ropinirole was evaluated by CCK-8 method. NS: no significance

**Fig. 2:**
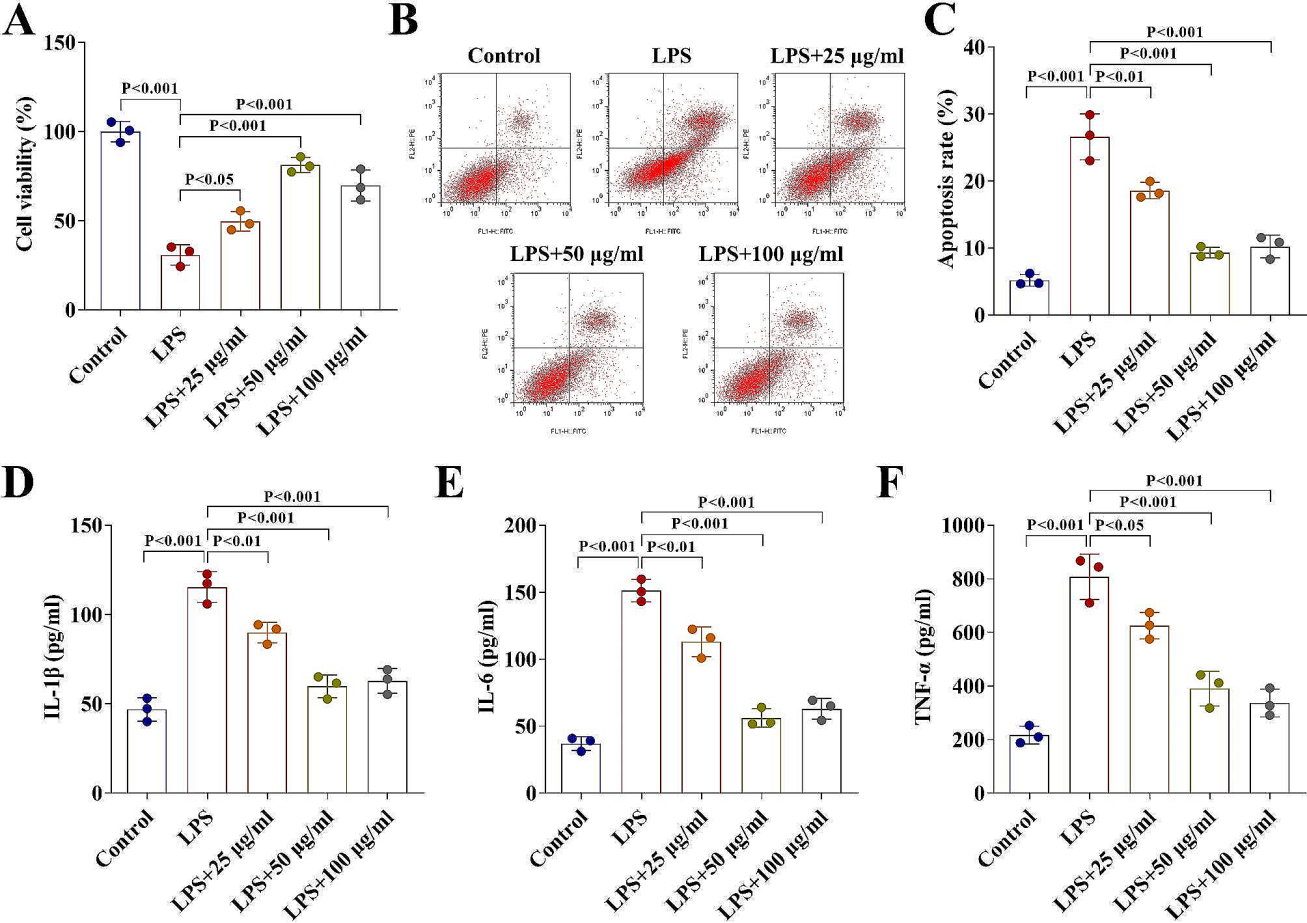
Ropinirole alleviates LPS-induced damage of HGFs. (**A**) Cell viability of HGFs treated with Ropinirole was evaluated by CCK-8 assay. (**B - C**) Flow cytometry assay was carried out to determine the cell apoptosis of HGFs. (**D - F**) Concentration of IL-1β, IL-6, and TNF-α in HGFs were detected by ELISA

First, we investigated the safe dose range of Ropinirole (Fig. 1A) for HGFs. As shown in Fig. 1B, the cell viability results suggested that the cell viability of HGFs was significantly inhibited when the concentration of Ropinirole exceeds 200 µg/ml, indicating that Ropinirole in this concentration range is toxic to HGFs. Ropinirole at concentrations of 25, 50 and 100 µg/ml had no statistically significant effect on the cell viability of HGFs (Fig. 1B). Subsequently, the protective effect of Ropinirole on LPS-induced HGF injury was studied. Cell viability was inhibited by LPS treatment in HGFs, and Ropinirole at concentrations of 25, 50 and 100 µg/ml significantly elevated the cell viability (Fig. 2A). LPS also promoted cell apoptosis and Ropinirole weakened the LPS effects by suppressing cell apoptosis (Fig. 2B − 2C). Meanwhile, inflammatory factors including IL-1β, IL-18, and TNF-α which were upregulated by LPS were significantly suppressed by Ropinirole (Fig. 2D − 2F). Ropinirole at a concentration of 50 µg/ml was close to that of 100 µg/ml in alleviating LPS-induced injury, so Ropinirole at a concentration of 50 µg/ml was used for follow-up experiments.

### Overexpression of NAT10 weakens the effects of Ropinirole on protecting HGFs

**Fig. 3:**
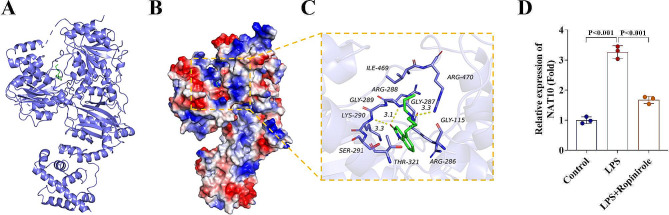
Molecular docking between active Ropinirole and NAT10. (**A - B**) The molecular docking between Ropinirole and NAT10. (**C - D**) NAT10 expression levels were detected by RT-qPCR in HGFs under the treatment of LPS and Ropinirole

**Fig. 4:**
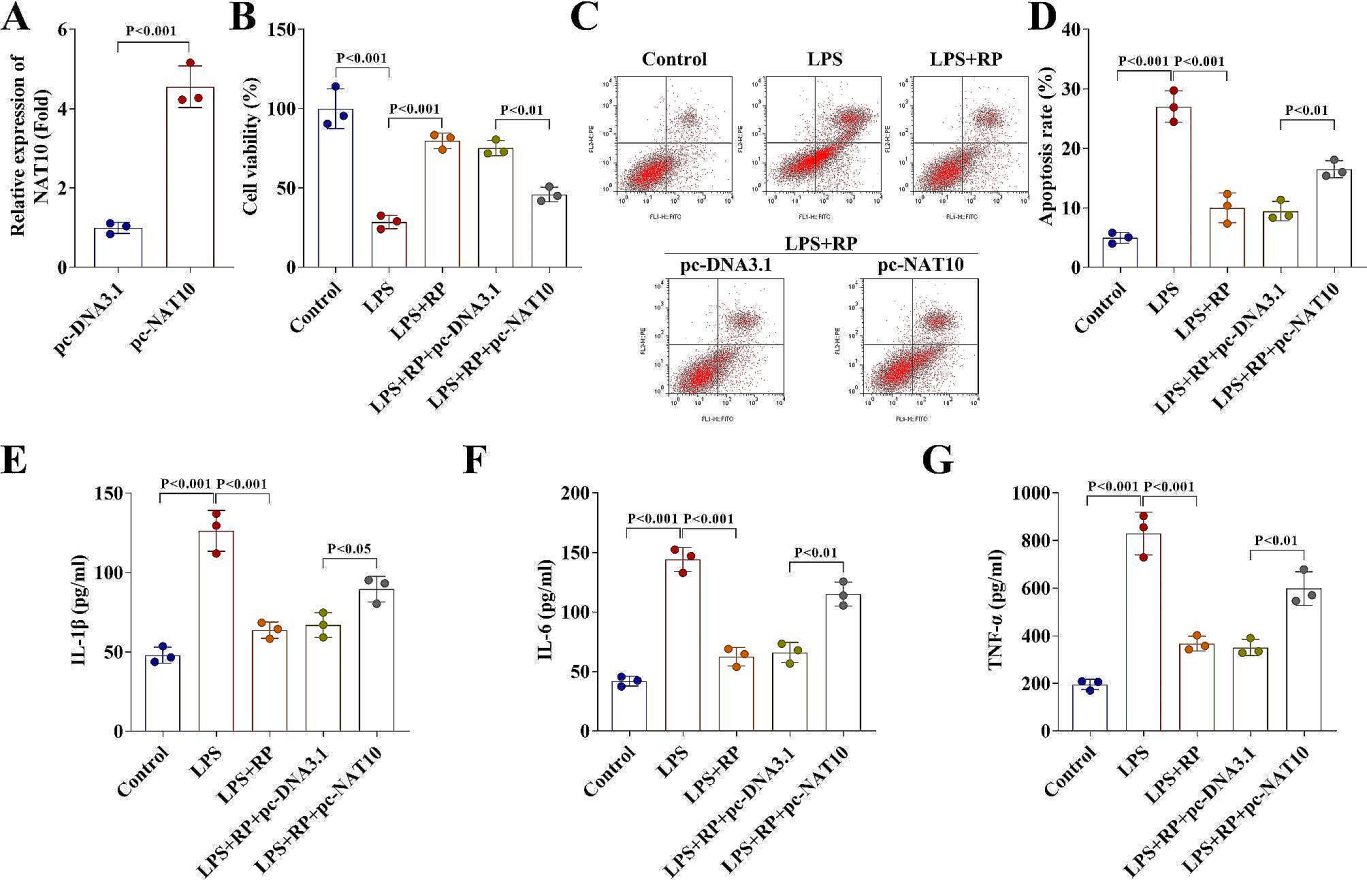
Overexpression of NAT10 weakens the effects of Ropinirole on protecting HGFs. (**A**) NAT10 expression levels were detected by RT-qPCR in HGFs before and after transfection. (**B**) Cell viability of HGFs treated with Ropinirole before and after transfection was evaluated by CCK-8 assay. (**C - D**) Flow cytometry assay was carried out to determine the cell apoptosis of HGFs treated with Ropinirole before and after transfection. (**E - G**) Concentration of IL-1β, IL-6, and TNF-α in HGFs treated with Ropinirole before and after transfection were detected by ELISA

Then the molecular docking results showed that Ropinirole can bind to NAT10 (Fig. 3A − 3B). As indicated in Fig. 3C, the mRNA levels of NAT10 was upregulated in LPS-treated HGFs, and was downregulated by Ropinirole treatment (Fig. 3D). The NAT10 expression was then successfully elevated in HGFs (Fig. 4A). Overexpression of NAT10 reversed the protective effects of Ropinirole on HGFs by inhibiting cell viability (Fig. 4B), increasing cell apoptosis (Fig. 4C − 4D), and upregulating levels of IL-1β, IL-18, and TNF-α (Fig. 4E − 4G).

### NAT10-mediated ac4C RNA acetylation promotes KLF6 mRNA stability

**Fig. 5:**
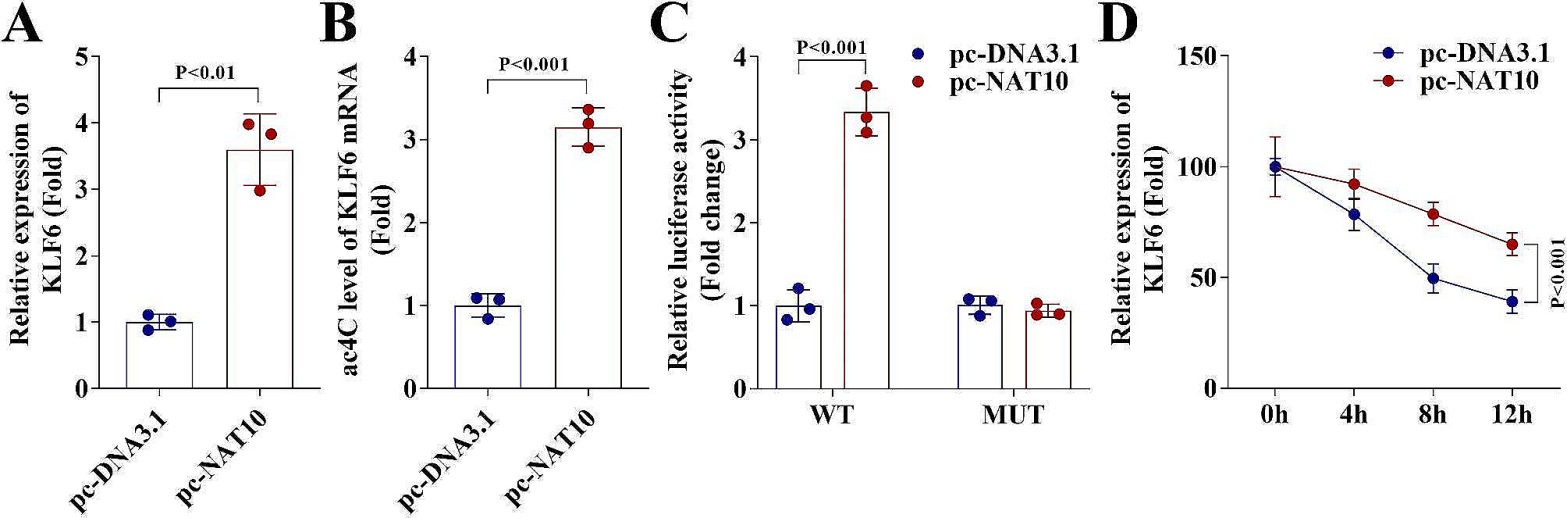
NAT10-mediated ac4C RNA acetylation promotes KLF6 mRNA stability. (**A**) RT-qPCR analysis of KLF6 mRNA levels in HGFs with overexpressed NAT10. (**B**) acRIP-PCR analysis of ac4C mRNA levels of KLF6 in HGFs with overexpressed NAT10. (**C**) The interaction relationship between NAT10 and KLF6 was verified by dual-luciferase reporter assay. (**D**) mRNA expression levels of KLF6 in HGFs transfected with overexpressed NAT10 vector after treatment with 2 mg/mL of actinomycin D for 0, 4, 8 and 12 h

KLF6 is involved in the regulation of physiological processes such as apoptosis and has been reported to enhance LPS-induced periodontal ligament cell damage [15]. Therefore, the NAT10-mediated ac4C modification of KLF6 was then investigated. Overexpression of NAT10 upregulated the mRNA levels of KLF6 in HGFs (Fig. 5A). The results of acRIP-qPCR suggested that ac4C mRNA levels of KLF6 were increased by NAT10 overexpression in HGFs (Fig. 5B). The interaction relationship between KLF6 and NAT10 was verified by dual-luciferase reporter assay (Fig. 5C). Afterwards, we assessed the stability of KLF6 mRNA in HGF and found that NAT10 overexpression enhanced the KLF6 transcript stability (Fig. 5D).

### Upregulation of KLF6 reversed the effects of NAT10 inhibition on HGFs

**Fig. 6:**
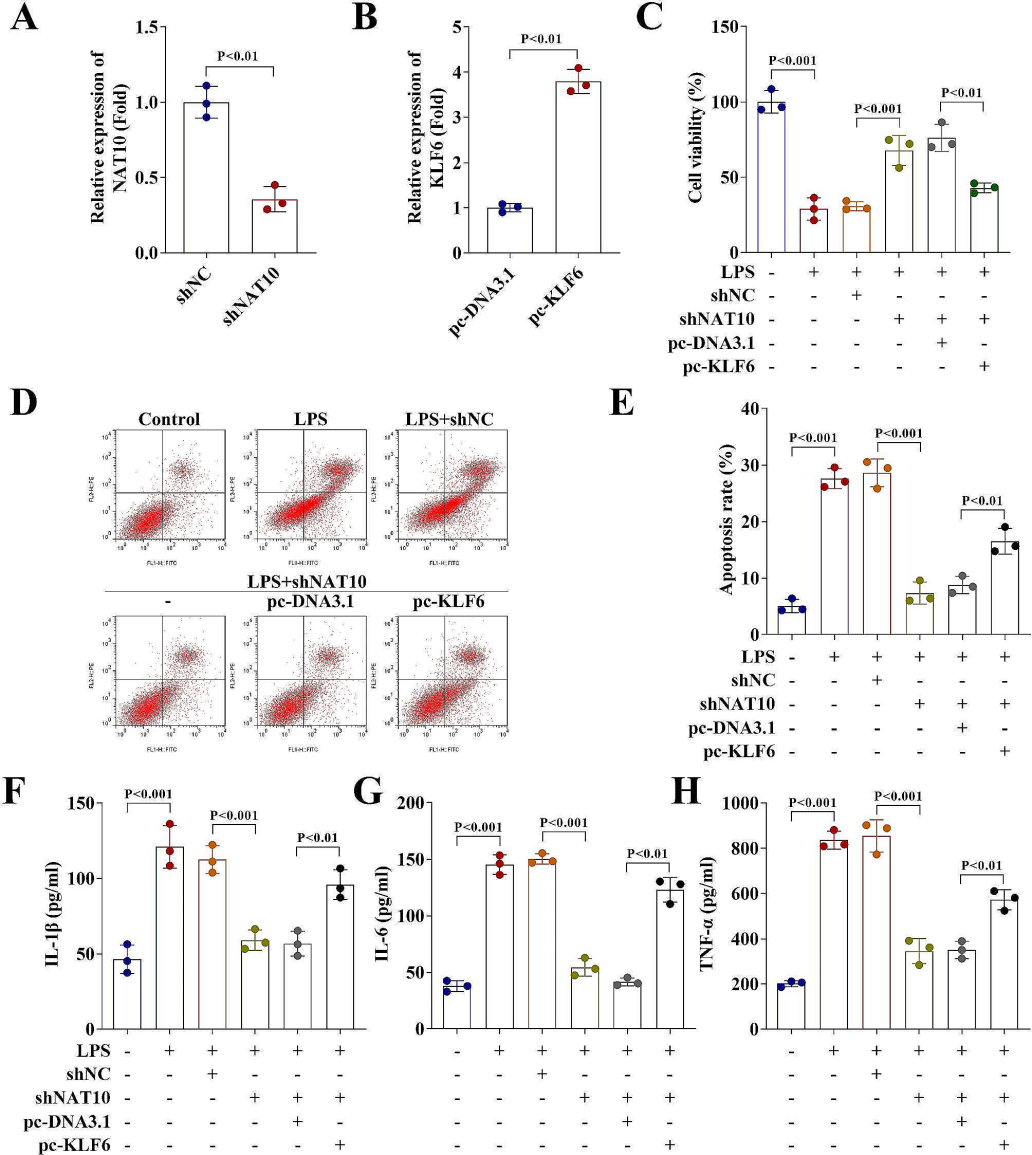
Upregulation of KLF6 reversed the effects of NAT10 inhibition on HGFs. (**A - B**) NAT10 and KLF6 expression levels in HGFs after transfection were detected by RT-qPCR. (**C**) Cell viability of HGFs treated with Ropinirole before and after transfection was evaluated by CCK-8 assay. (**D - E**) Flow cytometry assay was carried out to determine the cell apoptosis of HGFs treated with Ropinirole before and after transfection. (**F - H**) Concentration of IL-1β, IL-6, and TNF-α in HGFs treated with Ropinirole before and after transfection were detected by ELISA

Subsequently, the regulatory role of KLF6 on HGFs was investigated. The NAT10 and KLF6 expression were significantly downregulated and upregulated, respectively (Fig. 6A − 6B). Inhibition of NAT10 enhanced the effects of LPS on HGFs by promoting cell viability, inhibiting cell apoptosis, and downregulating levels of IL-1β, IL-18, and TNF-α. However, overexpression of KLF6 reversed the regulatory effects of NAT10 knockdown by inhibiting cell viability (Fig. 6C), promoting cell apoptosis (Fig. 6D − 6E), and elevating levels of IL-1β, IL-18, and TNF-α (Fig. 6F − 6 H).

## Discussion

The pathology of periodontitis is characterized by the destruction of periodontal supporting tissues, including the conjunctive epithelium, periodontal membrane and alveolar bone. How to reduce local inflammation and alveolar bone loss in patients with periodontitis is critical for regeneration and repair of alveolar bone. Previous studies have shown that periodontal tissue injury is mainly caused by immune inflammatory response generated by inflammatory mediators such as TNF-α, IL-1β and IL-6 released by the body. Therefore, in-depth study of the inflammatory signaling pathway and mechanism of periodontitis is helpful to accelerate the development of targeted periodontitis therapy and reduce the impact of periodontitis on systemic diseases.

There is rapidly growing evidence for the influence of inflammation on the development and progression of Parkinson’s disease, and Ropinirole has been reported to regulate inflammatory factors in serum of patients with Parkinson’s disease [16]. In this study, Ropinirole protected HGFs from LPS induced injury by suppressing inflammatory response, which is in line with the study of Isozaki et al. [10]. Moreover, Ropinirole can bind to NAT10 according to the the molecular docking results. NAT10 mRNA level has been found to be elevated in LPS-treated HGFs in this study, and Ropinirole significantly suppressed NAT10 levels.

NAT10 is the only gene found to catalyze the generation of ac4C modification in mRNA [17]. NAT10 can increase the stability of mRNA by catalyzing the generation of ac4C [18]. The content of ac4C in human body fluids changes significantly under disease conditions, such as gestational diabetes mellitus, interstitial cystitis, acquired immune deficiency syndrome, urogenital tract cancer and other diseases, the content of ac4C in urine of patients is significantly higher than that of healthy people [19–22]. Previous studies have shown that NAT10-mediated ac4C modification plays a regulatory role in various types of inflammation. For instance, NAT10 accelerates LPS-induced inflammation via the NOX2-ROS-NF-κB pathway in macrophages and Nox2 may be a potential target of NAT10 [13]. NAT10 mediated ac4C modification of ULK1 also regulates neutrophil pyroptosis in sepsis by activating STING pathway [23]. NAT10 promotes osteogenic differentiation of periodontal ligament stem cells by regulating VEGFA-mediated PI3K/AKT signaling pathway through ac4C modification [24]. Therefore, we speculated that NAT10 mediated ac4C modification may also participate in the regulation of inflammatory response in periodontitis. Our data suggested that overexpression of NAT10 weakened the protective effects of Ropinirole on HGFs. Therefore, the NAT10 mediated ac4C modification mechanism may be regulated by Ropinirole in periodontitis.

**Fig. 7:**
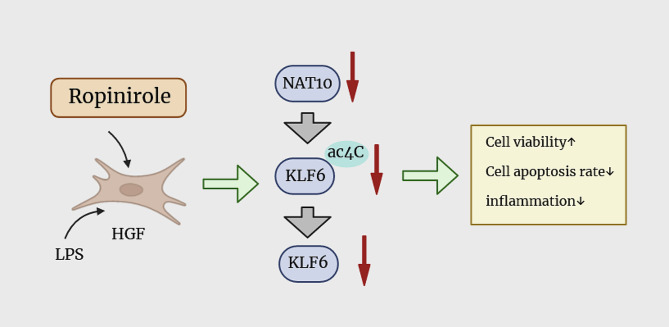
Graphical abstract

KLF6, a member of the KLF family, is a widely expressed nuclear transcriptional regulator [25]. KLF6 has established a promoting role in cell inflammation by working as the mediator of pro-inflammatory response [26–28]. Furthermore, miR-543-3p could down-regulate inflammation and inhibit periodontitis by targeting KLF6 [15]. Therefore, KLF6 may be a target for periodontitis treatment, and the interaction relationship between NAT10 and KLF6 in HGFs was investigated, and the results suggested that NAT10 can elevate the mRNA levels of KLF6 and the ac4C mRNA levels of KLF6. Meanwhile, the mRNA stability of KLF6 was also enhanced by NAT10. Therefore, Ropinirole protects HGFs by suppressing the NAT10 mediated ac4C modification of KLF6 from LPS injury (Fig. 7).

Our study tends to provide novel therapeutic treatment to relieve inflammation thereby for alveolar bone regeneration in periodontitis. However, there are still some limitations. Future studies should aim to enhance the possibility of Ropinirole as an adjunct drug in the treatment of periodontitis with validation through both clinical samples and animal models.

## Conclusion

LPS induced injury on HGFs is associated with the NAT10 mediated ac4C modification of KLF6, and Ropinirole exerts its protective effects on HGFs by inhibiting NAT10-ac4C-KLF6 axis. Our study suggested Ropinirole may be a promising anti-inflammatory agent for periodontitis treatment, and NAT10 and KLF6 may be potential targets to treat periodontitis.

## Data Availability

The datasets used and/or analysed during the current study are available from the corresponding author on reasonable request.
